# Controls on the Entrainment of Juvenile Chinook Salmon (*Oncorhynchus tshawytscha*) into Large Water Diversions and Estimates of Population-Level Loss

**DOI:** 10.1371/journal.pone.0101479

**Published:** 2014-07-14

**Authors:** Steven C. Zeug, Bradley J. Cavallo

**Affiliations:** Cramer Fish Sciences, Auburn, California, United States of America; University of Aveiro, Portugal

## Abstract

Diversion of freshwater can cause significant changes in hydrologic dynamics and this can have negative consequences for fish populations. Additionally, fishes can be directly entrained into diversion infrastructure (e.g. canals, reservoirs, pumps) where they may become lost to the population. However, the effect of diversion losses on fish population dynamics remains unclear. We used 15 years of release and recovery data from coded-wire-tagged juvenile Chinook Salmon (*Oncorhynchus tshawytscha*) to model the physical, hydrological and biological predictors of salvage at two large water diversions in the San Francisco Estuary. Additionally, entrainment rates were combined with estimates of mortality during migration to quantify the proportion of total mortality that could be attributed to diversions. Statistical modeling revealed a strong positive relationship between diversion rate and fish entrainment at both diversions and all release locations. Other significant relationships were specific to the rivers where the fish were released, and the specific diversion facility. Although significant relationships were identified in statistical models, entrainment loss and the mean contribution of entrainment to total migration mortality were low. The greatest entrainment mortality occurred for fish released along routes that passed closest to the diversions and certain runs of Chinook Salmon released in the Sacramento River suffered greater mortality but only at the highest diversion rates observed during the study. These results suggest losses at diversions should be put into a population context in order to best inform effective management of Chinook Salmon populations.

## Introduction

Diversion of freshwater for urban, industrial and agricultural use is a common practice around the world and is likely to become more frequent as demand increases [1]. There are numerous changes that take place in aquatic ecosystems as a result of flow reduction that can negatively affect fish including: alteration of sediment budgets, reduction or elimination of floodplain connectivity and altered cues for migration and reproduction [2], [3]. Additionally, fish may be lost through direct impingement on intake screens or entrainment into water storage facilities and canals [4]. Although many studies have documented responses of fish populations to altered flow regimes, ecological correlates of the entrainment process and population effects of direct loss at diversions are insufficiently documented and poorly understood [5].

Impingement and entrainment of large numbers of fishes have been reported in water diversions from rivers [6], [7], lakes [8] and estuaries [9], [10]. Most entrained fish are early life stages (age 0+) and species composition generally reflects habitat adjacent to the diversion [10]. Estimation of population impacts of diversion losses have been more difficult to quantify, although some such studies have been performed [11], [12]. Migratory fish species are unique in that their exposure to entrainment is primarily during periods of migration between habitats whereas resident species may be susceptible to entrainment until they leave the diversion vicinity or attain a less susceptible size [13].

Entrainment of juvenile anadromous salmonids (*Oncorhynchus spp*.) into two large water diversions in the San Francisco Estuary, California, USA has frequently been implicated in the decline of these species [14]. A portion of entrained salmon are salvaged and returned to the estuary however, mortality associated with the diversions is thought to impact these populations [15]. Loss densities (fish loss•volume of water diverted^−1^) at these diversions are currently used as triggers to restrict the volume of water diverted in an effort to protect endangered winter Chinook Salmon (*O. tshawytscha*), spring Chinook Salmon, and steelhead trout (*O. mykiss*). Loss density triggers can be problematic because they are not scaled for population abundance. Thus, triggers may be reached due to abundance fluctuations that do not represent an increase in the proportion of the population lost. In general, the physical and hydrological conditions associated with entrainment remain unclear and population-level effects of fish loss at the diversions are not known.

Our goals for this study were to elucidate these physical, biological and hydrologic conditions and to put entrainment losses in a population context. We assumed that salvage (the metric that can be measured) is proportional to total entrainment at the two diversions. To accomplish these goals we constructed statistical models of salvage and estimated total loss using 15 years of release and recovery data for coded wire tagged salmon raised at hatcheries throughout the Central Valley of California. The use of coded wire tagged fish is important relative to previous work because it allows loss to be scaled by the number of fish released; comparable analyses of raw salvage would be confounded by uncertainty in stock identity and population abundance. The results provide essential information for resource managers charged with recovering salmon stocks and implications for diversion losses in river systems worldwide.

## Methods

All data used in this study had previously been collected by state and federal resource agencies. The authors had no role in the handling of organisms.

### Study site

**Figure 1:**
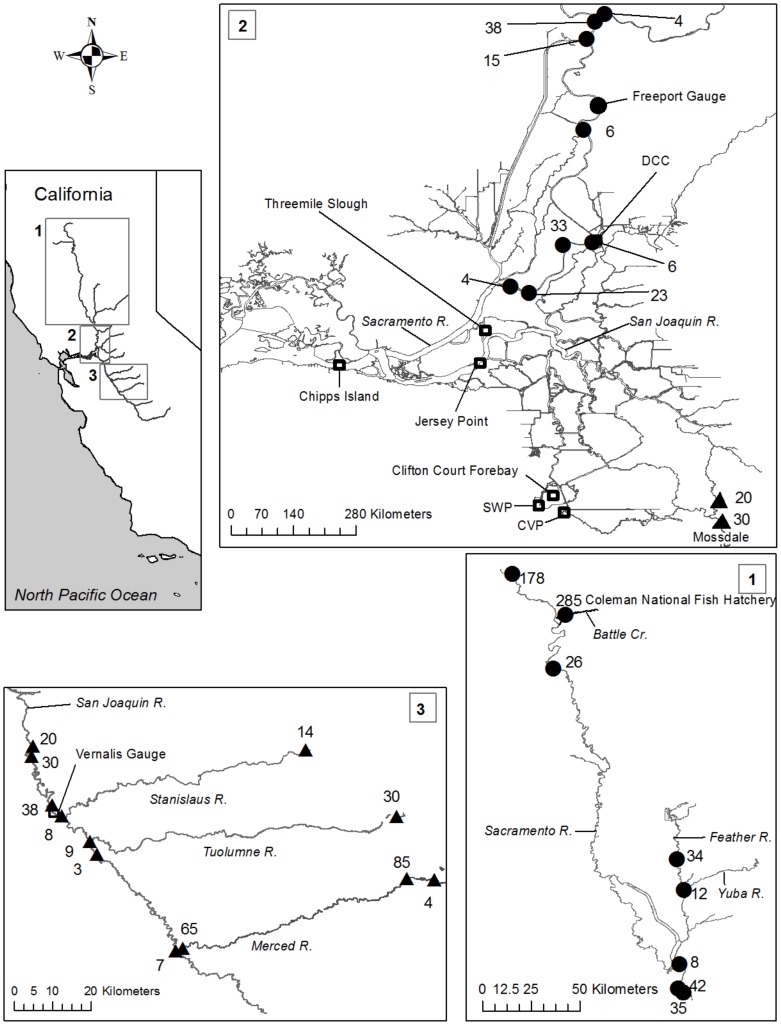
Map depicting the location of the study region within California and relevant locations within the study region. Release locations in the Sacramento River are indicated by closed circles and release locations in the San Joaquin River are indicated by closed triangles. The number of releases that occurred at each that location appears next to the marker. Abbreviations: SWP  =  State Water Project, CVP  =  Central Valley Project.

The Sacramento and San Joaquin Rivers drain approximately 40% of California' surface area including most of the western slope of the Sierra Nevada Mountains, the eastern slope of the Coast Range and portions of the southern Cascades. The two rivers converge in a tidal freshwater estuary known as the Sacramento-San Joaquin Delta (hereafter referred to as the “Delta”) before entering San Francisco Bay (Figure 1). Both rivers have been subjected to intense water development beginning in the late 19th century associated with urban and agricultural development in the Central Valley of California. Dam construction, channelization, levee construction and pollution have been prominent in both systems. Water diverted from these rivers provides water for millions of Californians and supports economically valuable agriculture throughout the Central Valley. Both rivers supported robust populations of Chinook Salmon in the past. However, 48% of historic habitat has been lost [16] and drastic reductions in the number of returning adults have triggered restrictions and even total closures of commercial and recreational fisheries in some years.

**Figure 2:**
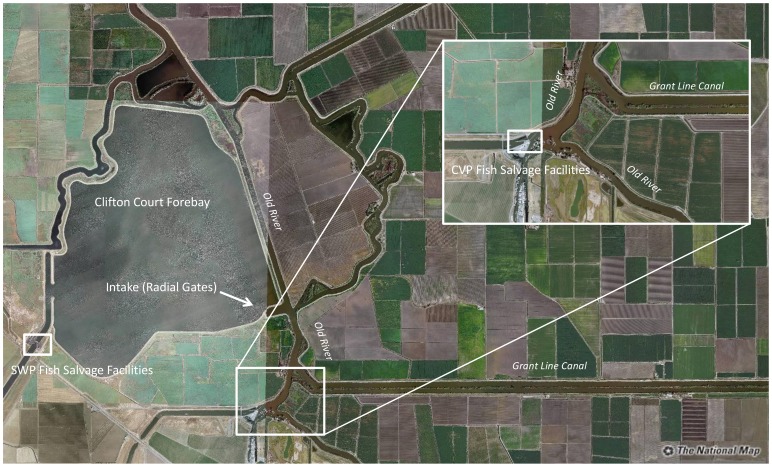
Aerial view of the layout of the two water diversion and fish salvage facilities. The State Water Project (SWP) diverts from Clifton Court Forebay that is filled from radial gates located on Old River; a distributary of the San Joaquin River. The Central Valley Project (CVP) diverts water directly from Old River. Image was downloaded from The National Map: http://nationalmap.gov/.

Freshwater is extracted in the tidal Delta at two large diversions that divert up to 60% of total flow in some years. Both diversions contain facilities where fish are salvaged and then released in the western Delta, away from the pumps. Fish entering the salvage facilities are subsampled at regular intervals (10–20 minutes•h^−1^) and total salvage is estimated based on the volume diverted and time since the previous sub-sample. Although salvage occurs at both diversions, there are significant differences in facility design that may affect the number of fish collected. The Central Valley Project (CVP) diverts water directly from a tidal channel in the Delta and fish are directed by a series of louvers into the salvage facility (Figure 2). The State Water Project (SWP) diverts water from a forebay filled by operable gates located on a tidal channel of the Delta (Figure 2). Thus, fish salvaged at the SWP have first been drawn into the forebay where they are exposed to resident predators before they are directed by louvers into the salvage facility as water is pumped out of the forebay. Additionally, the origin of salmon collected at the diversions is likely to have an influence on salvage. Fish released in the San Joaquin River are likely to first encounter the CVP whereas fish released in the Sacramento are likely to encounter the SWP first.

### Salmon releases

Chinook Salmon are raised at several hatcheries in the Sacramento-San Joaquin system and released at various locations as mitigation for habitat lost through dam construction, and as part of studies conducted by state and federal resource agencies. A portion of these hatchery fish have coded wire tags (CWTs) inserted for identification when recaptured. These tags are short lengths of steel wire with a numeric code that identifies a specific release group. Fish receiving a CWT also have their adipose fin clipped so tagged fish can be visually identified at capture. The tagging rate and number of fish released can vary considerably among runs. All hatchery winter run and late-fall run are tagged whereas the percentage of fall run tagged and released has varied between years. Spring run are raised at one hatchery; however, only 16 spring run release groups were identified within the study area and few of these fish ever arrived at salvage. Thus, spring run were not included in the analysis. Fish released into tributaries of the Sacramento River including: Battle Creek and Feather River are hereafter referred to as Sacramento River releases. Similarly, fish released into tributaries of the San Joaquin River including the Stanislaus River, Tuolumne River, and Merced River are hereafter referred to as San Joaquin River releases.

Juvenile salmon with an adipose clip collected at the diversions are retained, the coded wire tag is read, and the number of fish salvaged from that release group is estimated. Juvenile salmon exiting the Delta downstream of the diversions are sampled by a mid-water trawl at Chipps Island operated by the United States Fish and Wildlife Service (Figure 1). Trawling effort is variable among and within years and capture probability is low; however, some trawling occurs during all months of the year. Tagged salmon also are recovered from the commercial and recreational ocean fishery for several years after release.

Release data for juvenile salmon were obtained from the Regional Mark Processing Center coded wire tag database maintained by the Pacific States Marine Fisheries Commission (http://www.rmpc.org/). Data from release years 1993–2007 were queried from the database. We chose these years to represent current water management in the Delta which changed in the mid-1990's in response to the Bay-Delta Accord (California State Water Resources Control Board Ruling D-1641). Additionally, we excluded releases under 1000 individuals and releases made downstream of the last entrance to the interior Delta from the Sacramento River at Threemile Slough (Figure 1). The data queried included: release site, release size, date of release, mean fork length at release and age specific recoveries in the ocean. The number of salmon recovered in the ocean was expanded prior to analysis using the method described in Zeug and Cavallo [17]. Ocean recovery information was limited for later release years because the ocean fishery was restricted in 2007 and closed in 2008 in response to the collapse of the fall run. Recovery information was obtained from the United States Fish and Wildlife Service Chipps Island Survival table (http://www.fws.gov/stockton/jfmp/datamanagement.asp). These data included: number of tagged salmon recovered in the Chipps Island trawl, the expanded number of tagged salmon collected at the CVP and SWP salvage facilities, and the range of dates over which fish from each release group were captured in the trawl.

### Environmental data

Juvenile salmon are released in the Sacramento and San Joaquin rivers and tributaries (from <30 to >600 km from the diversions); however, they are not vulnerable to entrainment until they enter the tidal Delta. A study of salmon migration with acoustic telemetry indicated juvenile salmon migrated through the Delta in 6.4 days on average [18]. To capture the conditions experienced during Delta migration, hydrologic variables were averaged over 7 days after salmon entered the Delta. To estimate the date when each release group arrived at the Delta, we calculated the median date between the first and last capture in the Chipps Island trawl at the exit of the Delta. The 7 days prior to the median capture date was the time period over which hydrologic conditions were averaged.

Mean daily flow (hereafter “flow”) for the Sacramento River was obtained from the United States Geological Survey (USGS) gauge 11447650 at Freeport, California (Figure 1). San Joaquin River flow was obtained from USGS gauge 11303500 at Vernalis, California (Figure 1). Daily water diversion rates from the CVP and SWP were obtained from the DAYFLOW online data archive maintained by the California Department of Water Resources. An additional variable in the Sacramento River was the position of the Delta Cross Channel (DCC). The DCC is a large gate that diverts water from the main stem Sacramento River into interior portions of the Delta (Figure 1). When the gate is open, there is a greater probability of fish migrating down the Sacramento River will enter routes leading to the diversions [19].

### Data analysis

The response variable in all statistical models was the number of fish salvaged. The number of fish released was included as an offset variable to account for differences in release group size. Models were constructed separately for each diversion facility to determine if different independent variables affected salvage at diversions that extract water directly from a tidal channel (CVP) vs. a forebay (SWP). Models were also constructed separately for releases in the Sacramento and San Joaquin Rivers.

Independent variables in statistical models were selected based on hypothesized relationships with salvage. These variables could potentially affect the process of salvage or the exposure of fish to salvage. For example, zero salvage could occur because most fish were not exposed to entrainment or died prior to entering the Delta. Hypothesized relationships between independent variables and salvage are summarized in Table 1. To account for mortality prior to salvage, fork length at release and the shortest distance from release site to the nearest salvage facility were included. We expected survival would be negatively associated with distance [20] and positively associated with mean fork length [21]. For fish in the Delta we hypothesized that salvage would increase as flows decreased and as diversion increased. Previous analyses of fish entrainment have utilized a ratio of diversion to flow as a predictor of entrainment risk instead of using these variables as separate independent predictors. However, analyses of survival in the Delta have suggested diversion rate, and flow alone may have similar predictive capability without conflating these two variables [22]. To determine if a diversion-to-flow ratio was superior to modeling these effects separately, statistical models were constructed using both methods and then compared using Akaike's corrected information criteria (AIC_c_). When the difference between AIC_c_ values (ΔAIC_c_) for a pair of models was greater than 2.0, the model with the lower AIC_c_ values was considered to have the best support in the data.

**Table 1:** Predicted relationships between independent variables and salvage (count model) and independent variables and zero salvage (zero-inflation model).

Parameter	Count model	Zero-inflation model
Flow	−	+
Water diversion	+	−
DCC position	+	−
Fork length	−	+
Distance from salvage facilities	−	+
Chipps Island recoveries	−	+
Ocean recoveries	−	+

To account for fish that survived the Delta and avoided salvage, catch-per-unit effort in the Chipps Island trawl (number•min^−1^), and expanded ocean recoveries for each release were included in each model. We predicted that salvage would be negatively associated with recoveries at Chipps and in the ocean (i.e. when fewer fish are entrained at the diversion, more are available to be caught later in the trawl). Sacramento models also included a dummy variable for the position of the DCC where 1 =  open and 0 =  closed. All continuous variables were transformed into z-scores so results could be interpreted in units of standard deviations. A correlation analysis was performed to determine if multicollinearity existed among independent variables however, no strong correlations were identified.

Screening of the response variable indicated that many releases in both rivers resulted in zero salvage. Thus, zero-inflated negative binomial regression was employed. These models are composed of two parts; a count model that explains salvage as a function of covariates and a zero-inflation model that accounts for the processes that result in zero salvage as a function of covariates. The predicted sign of coefficients in the count model are listed in Table 1. Coefficients for the zero-inflation model would be predicted to have a sign that is opposite of the count model. Zero-inflated Poisson regression was explored but model diagnostics indicated over-dispersion. To determine if a zero-inflated model was necessary, a negative binomial regression model was constructed with the same independent variables and a Vuong non-nested hypothesis test was performed to determine if the zero-inflated model provided an improved representation of the data [23]. Once a model was identified, overall model fit was determined with a likelihood ratio test comparing an intercept-only model with the model containing independent variables. All modeling was performed with the R statistical program and the packages “pscl” and “MASS” [24].

To estimate a population-level effect of fish loss at the diversions, the contribution of loss relative to the total mortality rate during migration (hereafter referred to as relative loss) was estimated [11], [12]. Loss is defined as the fish that were entrained into the diversion and did not survive to release after salvage and trucking. To estimate loss for each release group, we first estimated the number of fish that encountered the louvers at each facility:
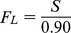
where *F_L_* is the number of fish that encountered the louvers, S is estimated salvage and louver efficiency is assumed to be 90% [15]. Total entrainment was then estimated as:
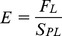
where *E* is total entrainment and *S_PL_* is the pre-louver survival. The pre-louver survival at the SWP was assumed to be 15%; the mean rate reported in a study by [25]. No data on pre-screen survival is available for the CVP so we assumed 85% following the methods of [15]. Total loss at each facility was then estimated as:
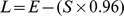
where *L* is total loss and survival during trucking and handling was 96% [15]. Loss estimates were summed for each facility and divided by the release group size to estimate the proportion of fish from each release group lost at the diversion.

To bracket the range of relative loss at the diversions, the highest and lowest observed mortality values during migration were used. Because published mortality estimates were not available for all release locations, only releases (n = 285) from Coleman National Fish Hatchery (CNFH) and directly into the tidal Delta (Sacramento River  = 129, San Joaquin River  = 88) were used. Separate estimates were calculated for each run. Though winter run were released upstream of the CNFH, we assumed that migration mortality of this run was similar to fish released directly from CNFH. Mortality estimates of Sacramento River releases during migration through the Delta were obtained from acoustic tagging experiments [19]. The highest through-Delta (Freeport to Chipps Island) mortality estimate from this study was 64.9% and the lowest was 45.7%. A single mean mortality estimate during migration from CNFH to Chipps Island (88%) was obtained from Michel [18] and San Joaquin estimates were obtained from Newman [26] and Buchanan et al. [27] where the highest through-Delta (Mossdale to Chipps Island) mortality estimate was 95.0% and the lowest was 79% (Mossdale to Jersey Point). The relative loss for each release was estimated as:
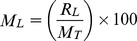
where *M_L_* is relative loss at the diversions, *R_L_* is the proportion of each release group lost at the diversions and *M_T_* is the total mortality during migration.

To quantify how uncertainty in the estimates of louver efficiency and pre-louver survival at both diversions influenced relative loss, a sensitivity analysis for the estimates of *M_L_* was performed using Monte Carlo methods. A distribution was constructed for each of the three estimates and they were allowed to vary one at a time while the other two were held constant. One hundred resamples were drawn, *M_L_* calculated, and the mean and standard deviation of the 100 resamples was estimated. The mean and standard deviation for pre-louver mortality at SWP [25] was used to inform a normal distribution. No data are available to inform a normal distribution for CVP pre-louver mortality so a uniform distribution was used where mortality ranged from 5–70%. A uniform distribution was also used for louver efficiency where values ranged from 50–95%.

There is considerable interest by resource managers in understanding how losses of salmon are related to diversion rate thus, the *M_L_* value for each release group was plotted against water diversion rate for each run of Chinook Salmon released from CNFH, and directly into the tidal Delta. These *M_L_* estimates were calculated with the lowest *M_T_* estimates to provide an estimate of the maximum mortality that could be accounted for by loss at the diversions. Additionally, relative loss was calculated assuming that no entrained fish were salvaged to estimate the effect of salvage facilities on loss estimates.

## Results

### Salvage of Sacramento River releases

A total of 749 releases comprised of >28•10^6^ fish were used to model salvage of Sacramento River Chinook Salmon. Fall run accounted for 419 releases, winter run 178 releases and late-fall run 152 releases. Only 16 release groups for tagged spring run Chinook Salmon were available and very few of these fish ever arrived at salvage; spring run Chinook Salmon were not included in further analyses. Across all Sacramento River releases an estimated 19281 CWT salmon were salvaged which represented 0.068% of the tagged fish released. Among the three runs of Chinook Salmon released, late-fall run fish were salvaged more frequently (0.2%) than winter and fall run (0.05 and 0.01% respectively). Average total loss (expanded for louver efficiency and pre-louver mortality) was greatest for late-fall run releases (0.84%) and lowest for fall run (0.03%) with an intermediate value for winter run (0.2%, Table 2).

**Table 2:** Means and coefficients of variation for variables used in models of salvage.

	Sacramento River			San Joaquin River
Parameter	Late-fall	Winter	Fall	Fall
Release size	58,365 (0.38)	6,354 (1.02)	43,936 (2.17)	24,917 (0.21)
Total salvage•release^−1^	118.5 (1.80)	2.7 (2.12)	1.9 (2.89)	149.6 (1.66)
Proportion salvaged	0.002 (1.64)	0.0005 (2.42)	0.0001 (2.96)	0.0058 (1.64)
Proportional loss	0.008 (1.65)	0.002 (2.57)	0.0003 (3.23)	0.014 (2.05)
Distance from salvage (km)	452 (0.47)	623 (0)	348 (0.61)	152 (0.56)
Flow m^3^•s^−1^	941 (0.83)	782 (0.55)	919 (0.69)	255 (0.90)
Water diversion m^3^•s^−1^	213 (0.50)	226 (0.30)	121 (0.74)	74 (0.56)
Salvage at CVP	44.4 (1.91)	1.1 (3.54)	0.5 (5.29)	102.5 (1.66)
Water diversion from CVP m^3^•s^−1^	97 (0.45)	105 (0.19)	54 (0.68)	40 (0.57)
Salvage at SWP	74.1 (1.84)	1.6 (2.34)	1.5 (3.26)	48.2 (2.14)
Water diversion from SWP m^3^•s^−1^	115 (0.59)	116 (0.31)	60 (0.93)	33 (0.75)
Length at release (mm)	128 (0.09)	78 (0.09)	67 (0.19)	82 (0.06)
Expanded ocean recoveries	593 (0.94)	16 (1.74)	365 (1.46)	85 (1.62)
Chipps trawl cpue	9.64 (0.98)	0.49 (1.40)	3.20 (1.57)	7.42 (1.38)

Variables were separated by run for Sacramento releases. Currently the San Joaquin only supports fall run Chinook Salmon.

A zero-inflated negative binomial model was a superior fit to the CVP salvage data for Sacramento River releases (V = 8.11, *P*<0.001), and the model fit the data well (likelihood ratio test, *P*<0.001). Similarly, salvage of Sacramento River releases at the SWP also was best described by a zero-inflated model (V = 7.66, *P*<0.001) and it was a good fit to the data (likelihood ratio test, *P*<0.001). The models that included flow and diversion as separate variables were a better fit to the CVP and SWP data than models using a ratio of diversion to flow with ΔAIC_c_ values of 23.4 and 76.9 respectively. The count models at both diversions revealed that there was a significant increase in salvage as diversion rate increased (Table 3). Contrary to expectations, salvage increased at both diversions when the DCC was closed. The DCC was only open for 48 of the 749 releases (6%) and given the large number of zeros in the data set, there was a lower probability of a large salvage event occurring when the DCC was open. Other significant relationships were specific to each facility. There was a significant negative relationship between flow and salvage, and a positive relationship between distance and salvage at the CVP facility. Fork length and Chipps Island recoveries had significant positive relationships with salvage at the SWP. There was also a significant negative relationship between ocean recoveries and salvage at the SWP (Table 3).

**Table 3:** Parameter estimates for zero-inflated negative binomial regression describing salvage of coded wire tagged juvenile salmon at the Central Valley Project and State Water Project facilities.

	Central Valley Project				State Water Project			
	Count model		Zero-inflation model		Count model		Zero-inflation model	
Parameter	estimate	*p-*value	estimate	*p-*value	estimate	*p-*value	estimate	*p-*value
Flow m^3^•s^−1^	**−0.651**	**<0.001**	**0.427**	**0.026**	**−0.208**	**0.227**	**0.521**	**<0.001**
Diversion m^3^•s^−1^	**0.663**	**<0.001**	**−0.443**	**0.004**	**0.29**	**0.005**	**−1.296**	**<0.001**
DCC open	**−0.800**	**0.001**	−0.134	0.751	**−0.917**	**<0.001**	−0.293	0.634
Fork length (mm)	0.008	0.944	**−1.497**	**<0.001**	**0.614**	**<0.001**	**−0.977**	**<0.001**
Distance from salvage facilities (km)	**0.331**	**0.001**	−0.246	0.116	0.149	0.085	0.144	0.270
Chipps Island recoveries (number•min^−1^)	0.194	0.067	−0.014	0.928	**0.345**	**0.011**	0.206	0.243
Ocean recoveries	0.020	0.827	−0.186	0.190	**−0.297**	**0.032**	−0.187	0.233

The count model describes the salvage process whereas the zero-inflated model describes the process resulting in zero salvage. All releases were in the Sacramento River.

The zero-inflation part of the analysis produced coefficients to estimate when salvage is zero versus any non-zero number. The zero-inflation models for Sacramento releases revealed consistent patterns between SWP and CVP. Specifically, there was a significantly greater likelihood of zero salvage when flows were higher, when water diversion was lower and when fish were released at a smaller size (Table 3). There were no significant relationships with DCC position in this portion of the model.

### Salvage of San Joaquin River releases

In the San Joaquin River there were 313 releases comprised of >7•10^6^ juvenile Chinook Salmon (Table 2). Only fall run were released in the San Joaquin River. A greater percentage of salmon released in the San Joaquin Basin were salvaged (0.6%) relative to any run of Sacramento River-origin fish. Mean total loss was also greater for releases in the San Joaquin River (1.4%, Table 2) relative to any run released in the Sacramento River. Similar to the Sacramento River releases, models that used diversion and flow as separate predictors were superior to models that used the diversion-flow ratio for the CVP and SWP (ΔAIC_c_  = 57.5 and 82.5 respectively).

A Vuong test indicated that a zero-inflated negative binomial model was the best description of San Joaquin releases salvaged at the CVP facility (V = 7.72, *P*<0.001). This model was a good fit to the data (likelihood ratio test, *P*<0.001). Additionally, a zero-inflated negative binomial model best represented the SWP salvage data (V = 6.22, *P*<0.001) and fit the data well (likelihood ratio test, *P*<0.001). The count models at both facilities yielded a significant increase in salvage as diversion rate increased (Table 4). The only other significant relationship in the count models at either facility was a positive coefficient for ocean recoveries in the SWP model.

**Table 4:** Parameter estimates for zero-inflated negative binomial regression describing salvage of coded wire tagged juvenile salmon at the Central Valley Project and State Water project facilities.

	Central Valley Project				State Water Project			
	Count model		Zero-inflation model		Count model		Zero-inflation model	
Parameter	estimate	*p-*value	estimate	*p-*value	estimate	*p-*value	estimate	*p-*value
Flow m^3^•s^−1^	0.098	0.247	−0.781	0.002	−0.019	0.874	−0.017	0.919
Diversion m^3^•s^−1^	0.453	<0.001	−0.791	<0.001	1.000	<0.001	−0.517	0.003
Fork length (mm)	0.030	0.692	−0.127	0.392	0.104	0.285	0.468	0.006
Distance from salvage facilities (km)	−0.060	0.505	0.091	0.567	−0.158	0.075	0.019	0.896
Chipps Island recoveries (number•min^−1^)	0.161	0.153	0.651	0.042	−0.295	0.054	0.171	0.503
Ocean recoveries	0.084	0.361	−1.996	<0.001	0.666	<0.001	−1.252	<0.001

The count model describes the salvage process whereas the zero-inflated model describes the process resulting in zero salvage. All releases were in the San Joaquin River.

The zero-inflation models for both facilities yielded significant negative relationships between the probability of zero salvage and diversion rate and ocean recoveries (Table 4). At the CVP, there was also a significant negative relationship between zero salvage and flow, and a significant positive relationship with recoveries at Chipps Island. For the SWP, fork length was found to have a significant positive relationship with zero salvage.

### Contribution to total migration mortality

Relative loss at the diversions was low for Sacramento River fish released at CNFH and directly into the Delta (Table 5). For CNFH releases, relative loss was greater at the SWP facility relative to the CVP facility although both values were <0.4%. A similar pattern was observed for Sacramento River fish released directly into the tidal Delta regardless of the migration mortality estimate. However, relative loss at the CVP was similar for fish released at CNFH and in the Delta whereas relative loss at the SWP was greater for fish released in the Delta. Mean relative loss of San Joaquin River releases was more than double that of Sacramento River releases at both facilities (Table 5). The pattern among the facilities was similar where relative loss was greater at the SWP relative to the CVP.

**Table 5:** Estimates of the % of total migration mortality accounted for by loss at each diversion (relative loss) for releases in the Sacramento and San Joaquin Rivers.

River	Release location	Facility	Migration mortality estimate (%)	Relative loss (%)	Confidence interval
Sacramento	CNFH	CVP	88.0	0.013	0.008–0.017
Sacramento	CNFH	SWP	88.0	0.372	0.328–0.416
Sacramento	Delta	CVP	64.9	0.009	0.004–0.013
Sacramento	Delta	CVP	45.7	0.012	0.006–0.018
Sacramento	Delta	SWP	64.9	0.449	0.237–0.661
Sacramento	Delta	SWP	45.7	0.614	0.324–0.905
San Joaquin	Delta	CVP	95.0	0.091	0.050–0.131
San Joaquin	Delta	CVP	79.0	0.109	0.060–0.157
San Joaquin	Delta	SWP	95.0	1.334	0.739–1.930
San Joaquin	Delta	SWP	79.0	1.596	0.884–2.309

Estimates were generated for Sacramento River fish released at Coleman National Fish Hatchery (CNFH) and directly into the tidal Delta. San Joaquin River estimates were only made for fish released into the tidal Delta.

**Figure 3:**
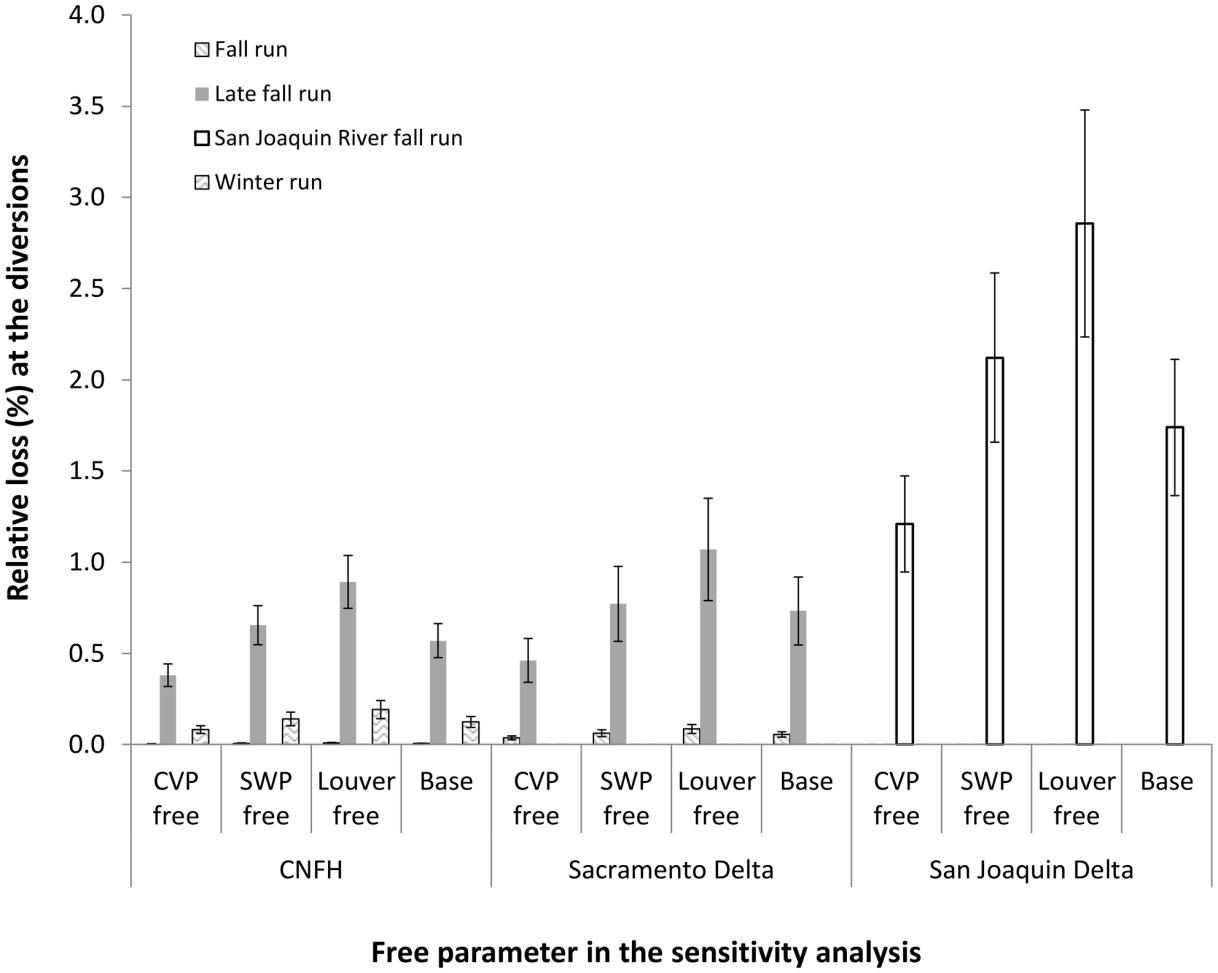
Results of the sensitivity analysis for the calculation of relative loss at the diversions as a function of uncertainty in the parameters used for the calculation. Three parameters (CVP pre-louver mortality, SWP pre-louver mortality and louver efficiency) were modeled as distributions and one parameter at a time was allowed to vary while the others were held constant. 100 re-samples were performed and the means and standard deviations are reported here.

The sensitivity analysis indicated that incorporating uncertainty in louver efficiency resulted in higher estimates of relative loss. Uncertainty in pre-louver survival at the CVP resulted in lower estimates relative to the baseline and uncertainty in pre-louver survival at the SWP produced similar estimates. The largest difference resulting from incorporation of parameter uncertainty was for San Joaquin River-origin fall run where mean estimates incorporating uncertainty in louver efficiency were 2.9% relative to the baseline value of 1.7%. All other differences were <1% (Figure 3).

**Figure 4:**
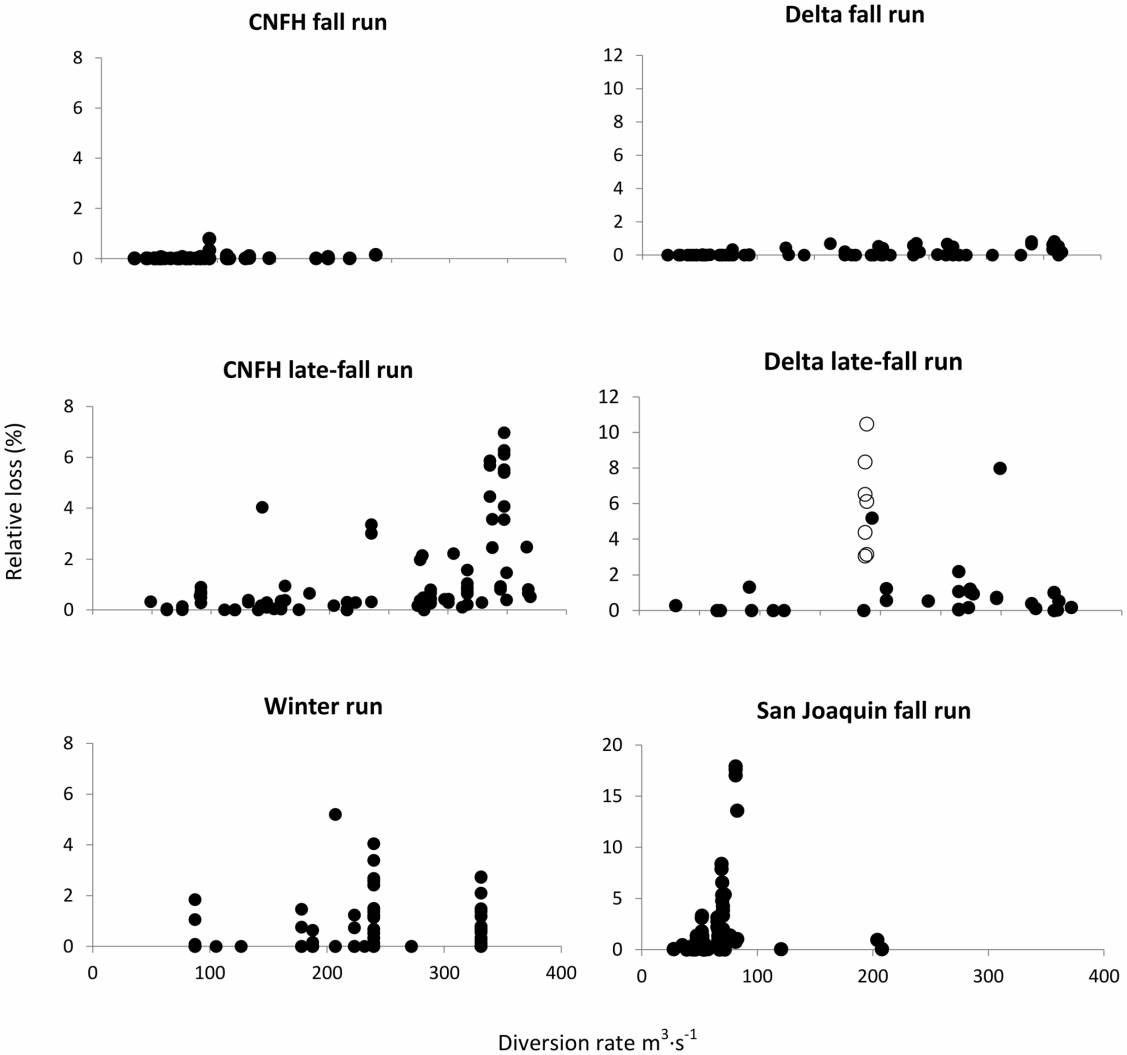
Plot of the percentage of migration mortality accounted for by loss at the two diversions (relative loss) as a function of diversion rate for three runs of Chinook Salmon released from the Coleman National Fish Hatchery (CNFH) or directly into the Delta. Open circles in the Delta late-fall run plot represent a set of releases that occurred within days of each other in 2007 and experienced unusually high loss. Note that the range of the y-axis changes among release locations.

For Sacramento River fall run Chinook Salmon, combined loss at the diversions (CVP + SWP) was always less than 1% of total migration mortality (relative loss) regardless of the diversion rate or release location (Figure 4). A small percentage of relative loss was observed for late-fall run released from CNFH until the diversion rate exceeded approximately 275 m^3^•s^−1^. Once this level of water diversion was reached, relative loss increased, although the variation also increased (Figure 4). Most late-fall Chinook Salmon released into the Delta experienced relative losses less than 2.5%. However, nine releases had relative losses that ranged between 3.0% and 10.5%. Seven of these releases occurred within days of each other in 2007 when the diversion rate was approximately 187 m^3^•s^−1^. Relative losses of winter run releases were variable throughout the range of observed diversion levels but were less than 2% for most releases and never exceeded 5.5% (Figure 4). Fall run Chinook Salmon released into the San Joaquin River experienced a greater relative loss at the diversions than any run released in the Sacramento River (Figure 4). Water diversion was less than 100 m^3^•s^−1^ during most San Joaquin River releases and although relative loss was less than 5% for most releases; this value ranged as high as 17.5%. Three releases occurred when the diversion rate was greater than 100 m^3^•s^−1^ and relative loss was less than 1% of total mortality for all three (Figure 4).

Salvage reduced relative loss by 19% for late-fall and winter run Chinook Salmon and 15% for fall run released at CNFH (Table 6). Salmon released in the Sacramento River received the greatest benefit from salvage with a reduction in relative loss of 42% and 41% for fall run and late-fall run respectively. Relative loss of fall run Chinook Salmon released in the San Joaquin River was reduced by 24% due to the presence of salvage facilities.

**Table 6:** Percent of migration mortality accounted for by loss at the diversions (relative loss) with and without accounting for salvage.

		Coleman Releases	Sacramento Delta Releases	San Joaquin Delta Releases
Fall	Salvage	0.017 (0.007)	0.076 (0.024)	1.704 (0.373)
	No salvage	0.020 (0.008)	0.132 (0.029)	2.242 (0.475)
Late-fall	Salvage	0.953 (0.153)	1.339 (0.430)	N/A
	No salvage	1.178 (0.189)	2.279 (0.535)	N/A
Winter	Salvage	0.222 (0.043)	N/A	N/A
	No salvage	0.273 (0.051)	N/A	N/A

## Discussion

During the study period, over 1000 releases of >35•10^6^ juvenile Chinook Salmon were performed. For releases in both rivers and among both diversions, there was a significant positive relationship between water diversion rate and salvage. The salvage facilities at these diversions have been likened to giant sampling devices [13]. Thus, it is not surprising that more fish are encountered as more water is sampled per-unit-time. Kimmerer [15] also found strong effects of water diversion on entrainment of salmon in this system and positive relationships between diversion volume and fish entrainment have been reported in other systems [7]–[9]. In contrast, the relationship between salvage and flow could not be generalized among rivers. For Sacramento River releases, there was a significant increase in salvage at the CVP facilities with decreasing flow. Supporting the same trend, greater flows significantly increased the probability of zero salvage of Sacramento River releases at both facilities. The lack of strong relationships between salvage and flow and the consistent strong relationships with salvage and diversion rate likely explain why using a ratio of diversion rate to flow where these two variables are conflated was a poor predictor relative to modeling these variables separately.

Perry [28] found that when discharge is low in the Sacramento River, flow changes direction with the tide at the junction of routes leading to the diversions and upstream flow increases the probability of salmon entering these junctions. Several releases of late-fall run in the Delta that were released within days of each other experienced unusually high rates of salvage. The timing of fish arrival at junctions leading to the diversions and tides were unmeasured here but may be important predictors of salvage and may have influenced these anomalous points in the late-fall Delta releases. For San Joaquin River releases, relationships with flow were less clear and the only significant relationship was between zero salvage at the CVP and flow. Salvage of San Joaquin River releases may only occur when fish are abundant near the diversion regardless of flow conditions. Other studies of fish entrainment at water diversions have found that catch is largely proportional to abundance in the channel being diverted from [6], [11].

Prior to constructing salvage models, we hypothesized that fish size at release and the distance of release sites from the diversions would influence how many fish would be susceptible to salvage through the effect of these variables on survival [20], [21]. However, both fish size and distance from the diversions were only strong predictors of zero salvage for releases in the Sacramento River. Zero salvage was more likely when fish were released at a smaller size and salvage of larger fish was greater at the SWP. Most potential predators of salmon smolts are gape-limited fishes such as striped bass (*Morone saxatilis*) and larger size may confer a survival advantage especially at the SWP where fish are exposed to high predation rates in the forebay prior to salvage [25]. Size effects were mostly insignificant for San Joaquin releases (although zero salvage was positively related to size at the SWP); however, fish were released at a wider range of sizes in the Sacramento River relative to the San Joaquin River. In particular, late-fall run Chinook Salmon were salvaged more frequently than any other run from the Sacramento River and this run was released at larger sizes than any other run.

The distance of release sites from the diversions also was important only for Sacramento River releases however the relationship was opposite of our expectation (positive coefficient). Large, late-fall run Chinook Salmon were salvaged more than any other run and these fish were primarily released at CNFH that was located 577 rkm from the closest salvage facility. There were no significant relationships between distance and salvage of San Joaquin River releases. The maximum distance of a release site in the San Joaquin was 262 rkm, which was less than half of the maximum distance of Sacramento releases (624 rkm) and may have masked distance effects in the San Joaquin River.

Relationships between salvage and recoveries downstream of the facilities were inconsistent and conflicting without any clear patterns. Previous studies in the Delta have attempted to link recovery rates at downstream locations with the water diversion rate from the Delta and have not produced strong evidence of an effect [26], [29], [30]. Additionally, Zeug and Cavallo [17] failed to find a relationship between salvage at these facilities and recovery rates in the ocean. Unlike many water diversions, these allow for a fraction of entrained fish to be returned to the channel alive and our results suggest that salvage reduced migration mortality due to entrainment by 15–42%.

Although several strong relationships were identified between salvage and predictor variables, total loss and the contribution of juvenile salmon loss at the diversions to total mortality (relative loss) during migration was low. This may partially explain the poor and inconsistent relationships between salvage and recovery of tagged fish in Chipps trawl and the ocean. Although diversion-related entrainment is frequently invoked as a threat to fish populations, few studies have evaluated population-level effects of fish loss at diversions [5]. Several studies of entrainment loss relative to population mortality have reported relatively small contributions of entrainment similar to the estimates reported here [9], [11], [12]. The location of the diversions may also contribute to low relative loss. Both diversions are located on a distributary of the San Joaquin River thus, only salmon migrating through that route are susceptible to entrainment. In general, less than half of the juvenile salmon migrating down the San Joaquin River are likely to enter channels leading to the diversions [27] and even fewer Sacramento River-origin salmon are likely to enter this channel [19].

Although the results presented here suggest the total effect of loss at these diversions on migrating juvenile salmon is small, caution should be used when applying these results to other systems or even to the Sacramento-San Joaquin Delta generally. First, these diversions include salvage facilities that allow some fish to be returned alive. In many systems, fish that are entrained in diversions cannot return and are lost. Although it is largely unknown how these losses affect populations, Roberts and Rahel [4] suggested these diversions can function as sink habitats. Second, there are a large number of small diversions in the Delta that do not contain fish screens or salvage mechanisms and the aggregate effect of these diversions could be significant [31]. The calculation of entrainment loss includes estimates for louver efficiency and pre-louver survival that have low certainty and better quantitative estimates for these parameters could reveal greater estimates of total loss. Finally, all fish in this study (and in the acoustic studies used to calculate migration mortality) were hatchery reared fish. Thus, we are making the assumption that the behavior and survival of hatchery Chinook Salmon is similar to naturally produced fish in both rivers.

The data presented here indicated that a variety of hydrologic (diversion rate and flow), physical (distance from facilities) and biological (fish size) factors influence the salvage of CWT juvenile Chinook Salmon at two large water diversions. However, the relative importance of these factors varied among the two river systems where fish were released and among the two diversions which differed in the configuration of water diversion. Attempts to increase survival of juvenile salmon migrating through the Delta have largely focused on restriction of water diversion [14]. Yet the total contribution of loss at these facilities was small relative to total migration mortality and relationships between salvage and recoveries downstream were inconsistent and conflicting. The ability of fish to be salvaged and the physical location of the pumps off of the main stem rivers likely reduced the total population-level effect of these diversions. As water development continues worldwide, the inclusion of effective salvage facilities in diversion designs, and careful selection of diversion locations could help mitigate fish losses.
